# A prototype RT-PCR assay for detection of XMRV in multiple human sample types

**DOI:** 10.1186/1742-4690-8-S1-A220

**Published:** 2011-06-06

**Authors:** Ning Tang, Andrea Frank, Robert Kowal, Gregor Leckie, John Hackett, Graham Simmons, Michael Busch, Klara Abravaya

**Affiliations:** 1 Abbott Molecular Inc., Des Plaines, IL, 60018, USA; 2 Abbott Diagnostics, Abbott Park, North Chicago, Illinois, 60064, USA; 3 Blood Systems Research Institute, University of California, San Francisco, CA, 94118, USA

## Background

Xenotropic murine leukemia virus-related virus (XMRV) has been reported to be associated with prostate cancer and chronic fatigue syndrome. To help resolve the role of XMRV in human disease, it is critical to develop sensitive and accurate PCR assays for XMRV detection.

## Materials and methods

The automated m2000 system RT-PCR assays detect the pol and env regions of XMRV in whole blood, plasma, urine, and cell pellets. Assay performance was assessed by testing two blinded panels prepared by the Blood XMRV Scientific Research Working Group (SRWG), as well as clinical specimens.

## Results

For the SRWG whole blood panel, all six XMRV negative samples were assay negative, while all the 22Rv1-spiked samples from 0.5 cells/mL to 9,900 cells/mL were detected (3/3 for each panel member). For the SRWG plasma panel, all six XMRV negative samples were assay negative, while the 22Rv1 supernatant-spiked plasma samples were detected as positive, 1/3 for the 3.2 XMRV copies/mL panel member, 2/3 for the 16 copies/mL panel member, and 3/3 for panel members containing > 80 copies/mL. For screening of clinical specimen, assay positive results were obtained for 10% (2/20) of prostate cancer FFPE specimens, 0.5% (2/400) of prostate cancer urine pellets, and 0% (0/135) of the cervical swab specimens.

## Conclusions

We developed an automated high throughput real-time RT-PCR assay (with internal control) to detect XMRV. Use of this assay should assist in elucidation of the role of XMRV in human disease.

